# Supplementary material for: mGluR5 is transiently confined in perisynaptic nanodomains to shape synaptic function
Source: Nat Commun. 2023 Jan 16;14:244. doi: 10.1038/s41467-022-35680-w (PMC9842668; doi:10.1038/s41467-022-35680-w)
Supplement: Supplementary file 1 — Supplementary Information [file 41467_2022_35680_MOESM1_ESM.pdf]

SUPPLEMENTARY INFORMATION

mGluR5 is transiently confined in perisynaptic nanodomains to shape synaptic function

Nicky Scheefhals, Manon Westra and Harold D. MacGillavry

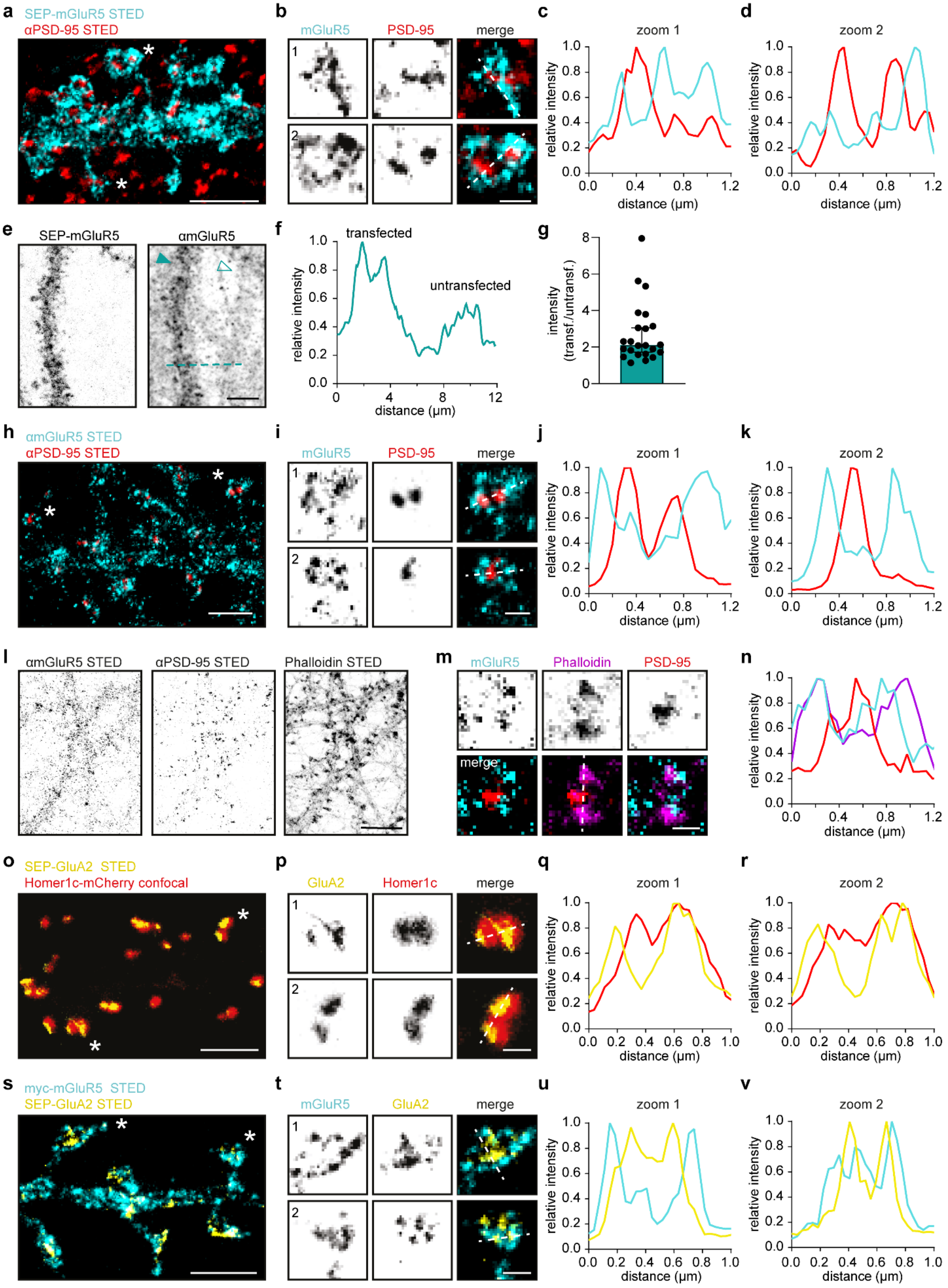
a
SEP-mGluR5 STED
αPSD-95 STED
b
mGluR5
PSD-95
merge
c
zoom 1
d
zoom 2
relative intensity
distance (µm)
e
SEP-mGluR5
αmGluR5
f
transfected
untransfected
g
intensity
(transf./untransf.)
h
αmGluR5 STED
αPSD-95 STED
i
j
k
l
αmGluR5 STED
αPSD-95 STED
Phalloidin STED
m
mGluR5
Phalloidin
PSD-95
n
o
SEP-GluA2 STED
Homer1c-mCherry confocal
p
GluA2
Homer1c
q
r
s
myc-mGluR5 STED
SEP-GluA2 STED
t
u
v

Figure S1. mGluR5 is largely excluded from the synapse

(a) Representative two-color gSTED image of dendrite with SEP-mGluR5 expression, with anti-GFP staining (cyan; Atto647N) to label surface-expressed receptors and co-stained for anti-PSD-95 (red; Alexa594). Scale bar, 2 μm. (b) Zooms of dendritic spines indicated in *a* with asterisks. Scale bar, 500 nm. (c) Line profile of spine 1 and (d) spine 2, indicated with dotted line in *b*. (e) Representative confocal images of total mGluR5 levels in SEP-mGluR5 transfected and untransfected neurons (anti-mGluR5 Millipore Alexa594). Scale bar, 5 μm. (f) Line profile of anti-mGluR5 in a SEP-mGluR5 transfected dendrite (filled arrowhead) and an untransfected dendrite (open arrowhead), indicated with dotted line in *e*. (g) Quantification of the ratio of mGluR5 intensity in transfected over untransfected neurons (n = 23). Data is represented as median with 95% CI. (h) Representative two-color gSTED image of dendrite endogenously stained for total mGluR5 (cyan; anti-mGluR5 Millipore Atto647N) and PSD-95 (red; Alexa594). Scale bar, 2 μm. (i) Zooms of dendritic spines indicated in H with asterisks. Scale bar, 500 nm. (j) Line profile of spine 1 and (k) spine 2, indicated with dotted line in *i*. (l) Representative three-color gSTED image of dendrite endogenously stained for total mGluR5 (cyan; anti-mGluR5 Alomone Atto647N), Phalloidin (magenta; Alexa594) and PSD-95 (red; Alexa488). Scale bar, 10 μm. (m) Zoom of dendritic spine stained for mGluR5 (cyan), Phallodin (magenta) and PSD-95 (red). Scale bar, 500 nm. (n) Line profile of spine, indicated with dotted line in *m*. (o) Representative gSTED image of dendrite expressing SEP-GluA2, additionally labeled with anti-GFP nanobody Atto647N to visualize surface-expressed receptors (yellow), and Homer1c-mCherry (red; confocal). Scale bar, 2 μm. (p) Zooms of dendritic spines indicated in *o* with asterisks. Scale bar, 500 nm. (q) Line profile of spine 1 and (r) spine 2, indicated with dotted line in *p*. (s) Representative two-color gSTED image of dendrite expressing myc-mGluR5 (cyan; anti-myc Alexa594 surface labeling) and SEP-GluA2 (yellow, anti-GFP Atto647N surface labeling). Scale bar, 2 μm. (t) Zooms of dendritic spines indicated in *s* with asterisks. Scale bar, 500 nm. (u) Line profile of spine 1 and (v) spine 2, indicated with dotted line in *t*. All experiments in this figure were replicated in cultures from at least 2 independent preparations of hippocampal neurons. Source data are provided as a Source Data file.

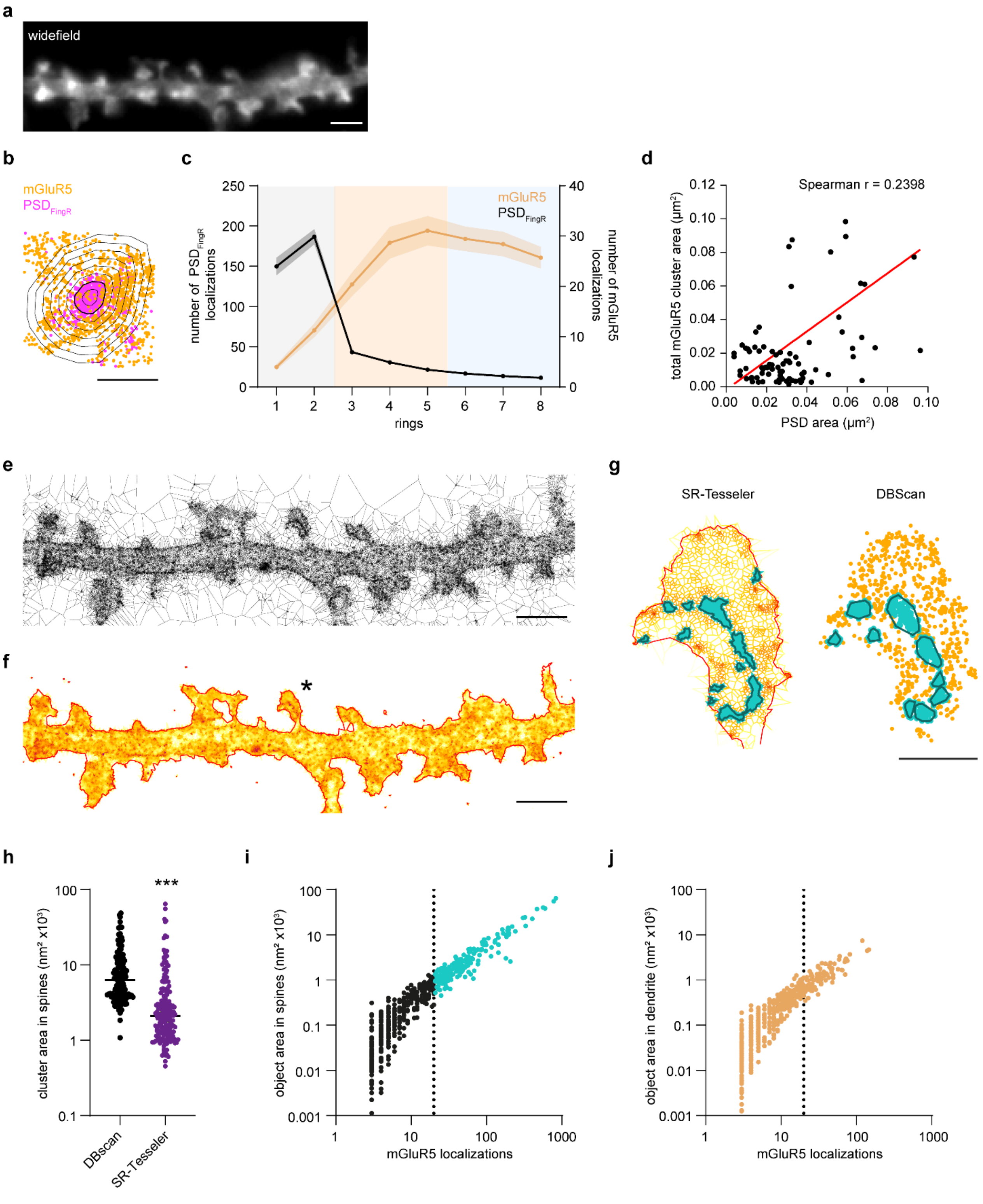
a
widefield
b
mGluR5
PSD FingR
c
mGluR5
PSD FingR
number of PSD FingR localizations
number of mGluR5 localizations
rings
d
Spearman r = 0.2398
total mGluR5 cluster area (µm²)
PSD area (µm²)
e
f
*
g
SR-Tesseler
DBScan
h
***
cluster area in spines (nm² x10³)
DBscan
SR-Tesseler
i
object area in spines (nm² x10³)
mGluR5 localizations
j
object area in dendrite (nm² x10³)
mGluR5 localizations

Figure S2. mGluR5 is enriched in distinct perisynaptic nanodomains

(a) Widefield of dendrite expressing SEP-mGluR5 and PSD_{FingR}-mEos3.2 (green channel). Scale bar, 2 µm. (b) The rings from *Figure 2e* with the corresponding localizations of mGluR5 (orange) and PSD_{FingR} (cyan). Scale bar, 500 nm. (c) Absolute number of PSD_{FingR} (black; plotted on left y-axis) and mGluR5 (orange; plotted on right y-axis) localizations in rings 1 to 8. Data are represented as means ± SEM. (d) Correlation between total mGluR5 cluster area and PSD area (in μm^2) (Correlation Spearman r = 0.24, p = 0.03). (e) Voronoï based segmentation performed with SR-Tesseler of the same dendrite shown in *Figure 2a*. Scale bar, 2 µm. (f) Local density map created from the Voronoï diagrams, the red line represents the neuron outline. Scale bar, 2 µm. (g) Zooms of the dendritic spine indicated in *f* with an asterisk, showing the Voronoï polygons with detected clusters (cyan) using SR-Tesseler (left) and mGluR5 localizations belonging to clusters (cyan) detected using DBScan (right). Scale bar, 500 nm. (h) Quantification of median mGluR5 cluster area in spines as determined by DBScan and SR-Tesseler, shown in log 10 scale (n = 18 spines, p < 0.0001, two-sided Mann-Whitney test). Data are represented as median with 95% CI. (i) mGluR5 object area plotted as a function of number of localizations in spines and (j) dendrites determined using SR-Tesseler, shown in log 10 scale. The dotted line at 20 localizations indicates the cut-off of minimum number of locaizations per cluster otherwise set as criteria in the DBScan and SR-Tesseler analysis, and the cyan scatters are included for analysis in *h*. Source data are provided as a Source Data file.

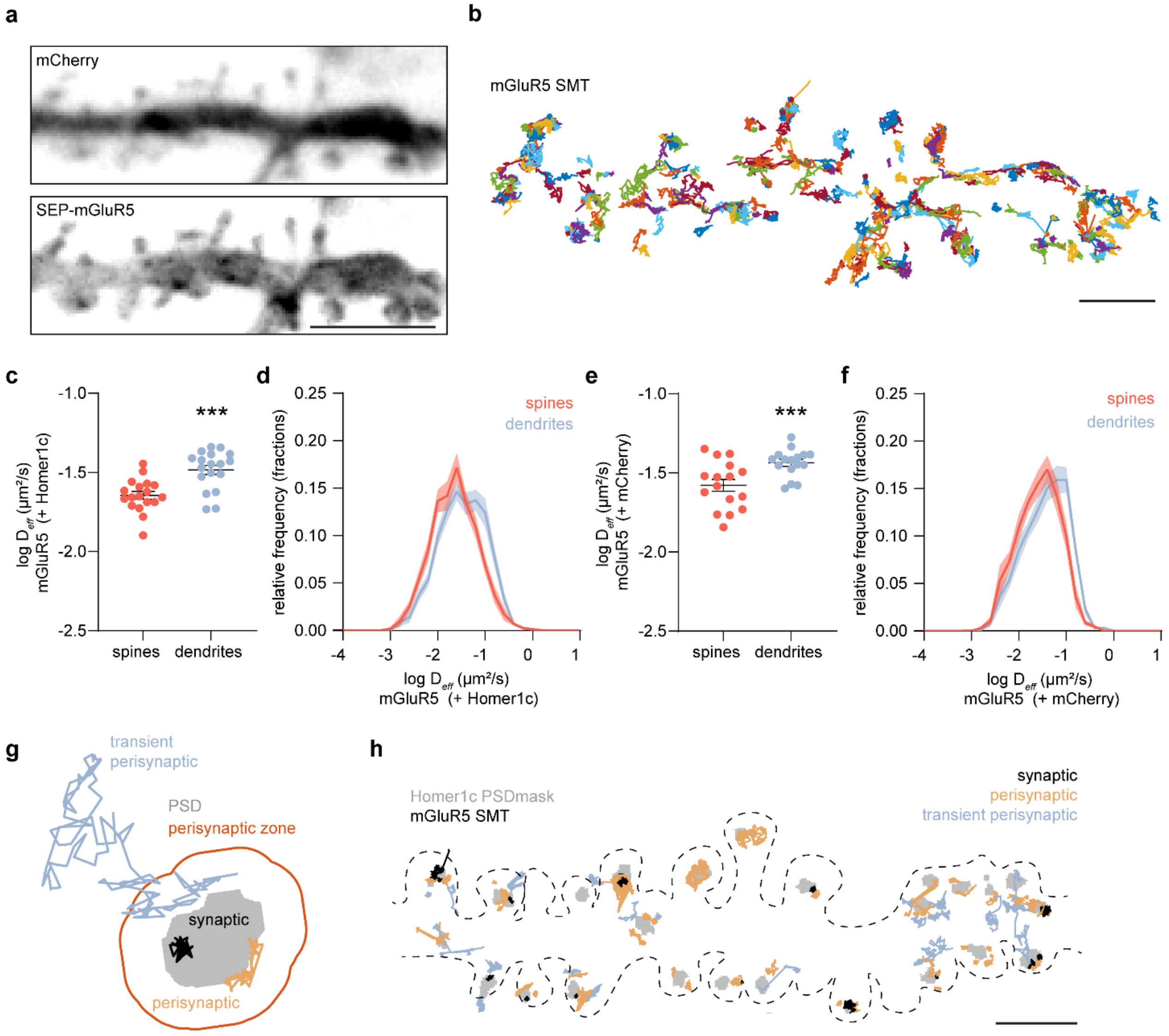

Figure S3. Single-molecule tracking resolves the subsynaptic dynamics of mGluR5

(a) Widefield of dendrite expressing mCherry and SEP-mGluR5. Scale bar, 5 µm. (b) SMTs of mGluR5 (random colors) in the same dendrite as shown in *a*. Scale bar, 2 µm. (c) Mean log D_{eff} per neuron ($p < 0.0001$, two-sided paired t-test) and (d) relative frequency distributions of D_{effs} of mGluR5 trajectories in spines and dendrites co-transfected with Homer1c-mCherry (n = 18). (e) Mean log D_{eff} per neuron ($p = 0.0006$, two-sided paired t-test) and (f) relative frequency distributions of D_{effs} of mGluR5 trajectories in spines and dendrites co-transfected with mCherry (n = 16). (g) mGluR5 trajectories are assigned to different categories based on the degree of colocalization with the PSD (grey) and perisynaptic zone (orange): synaptic trajectories (black; ≥80 % overlap with PSD), perisynaptic trajectories (orange; ≥60 % overlap with the perisynaptic zone and <80 % overlap with PSD) and transient perisynaptic trajectories (blue; >0 %, but <60 % overlap with the perisynaptic zone). (h) Synaptic (black), perisynaptic (orange) and transient perisynaptic (blue) SMTs of mGluR5 relative to the Homer1c PSD mask (grey) in the dendrite shown in

Figure 3a and b. This dendrite is an example of the mGluR5 trajectories that are included for analysis, compared to the same dendritic stretch in *Figure 3b* with all obtained trajectories shown. Scale bar, 5 µm. Data are represented as means ± SEM. ***$p < 0.001$. Source data are provided as a Source Data file.

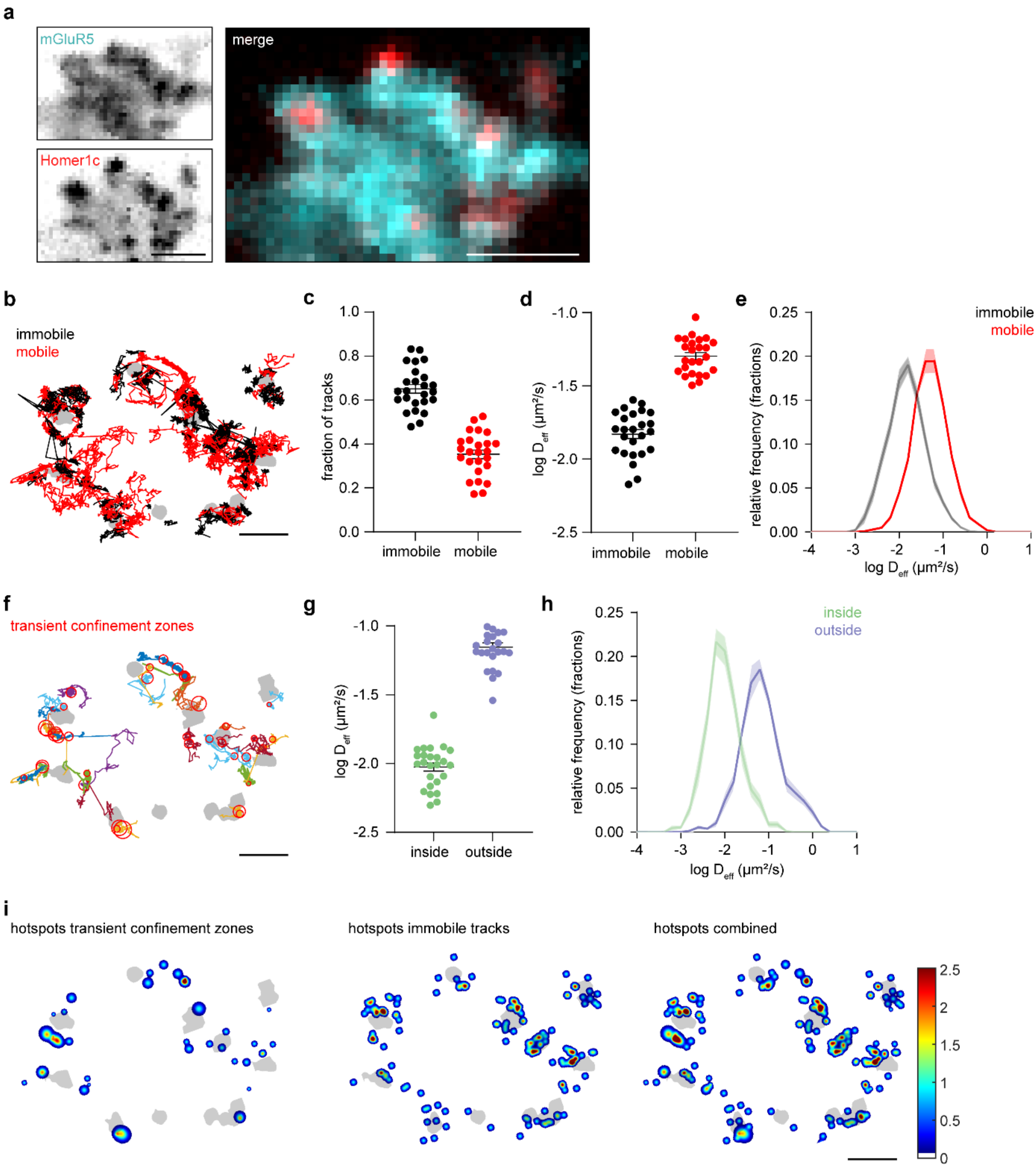

Figure S4. mGluR5 is not stably anchored, but dynamically exchanges at perisynaptic nanodomains (a) Widefield image of a dendrite expressing SEP-mGluR5 (cyan) and Homer1c-mCherry (red). Scale bar, 2 µm. (b) The same dendrite, with SMTs color-coded for immobile (black) and mobile (red) mGluR5 relative to the Homer1c PSD mask. Scale bar, 1 µm. (c) Fraction of immobile and mobile mGluR5 trajectories (n = 25 neurons). (d) Mean log D_{eff} per neuron and (e) relative frequency distributions of D_{effs} of immobile and mobile mGluR5 trajectories (n = 25). (f) Transient confinement zones (red circles) of the mobile mGluR5

trajectories (random colors) shown in *b*. Scale bar, 1 µm. (g) Mean log D_{eff} per neuron and (h) relative frequency distributions of D_{effs} of mGluR5 trajectories inside (green) and outside (blue) confinement zones (n = 25). (i) Hotspots of transient confinement zones (left), immobile track centers (middle) and both images combined (right), color-coded for the frequency of confinement zones and/or immobile tracks. Scale bar, 1 µm. Data are represented as means ± SEM. Source data are provided as a Source Data file.

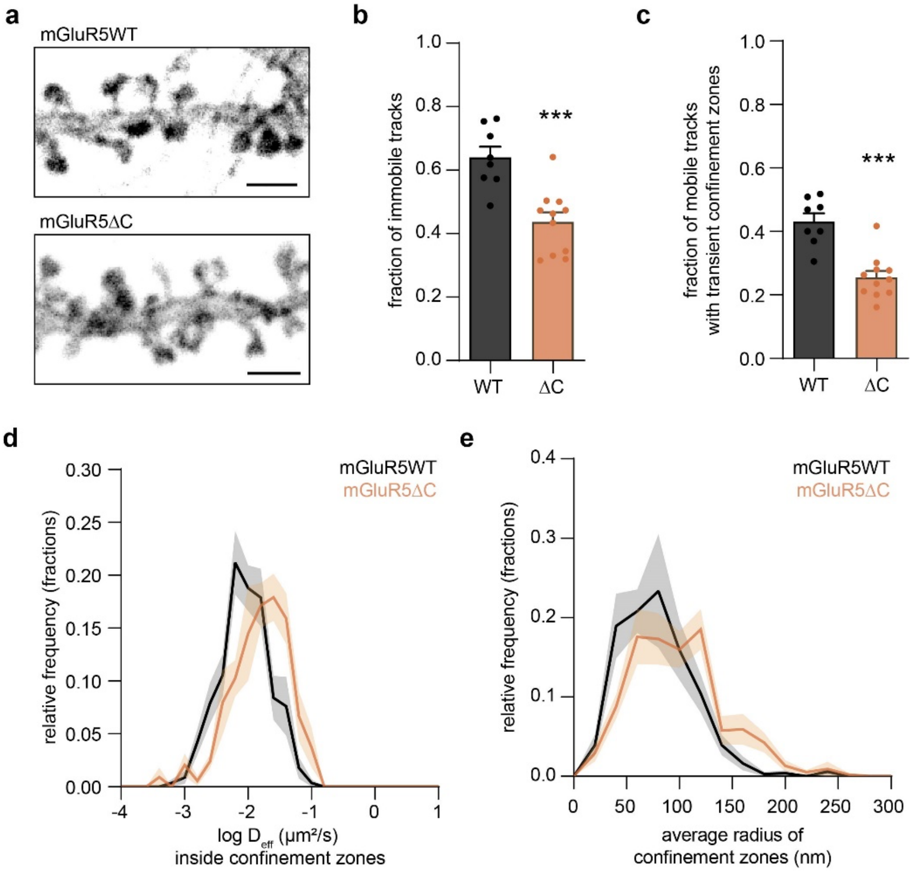

Figure S5. The C-terminal domain of mGluR5 regulates transient confinement

(a) Representative confocal images of dendrite with SEP-mGluR5WT (top) and SEP-mGluR5ΔC (bottom) expression, surface-labelled with an anti-GFP nanobody Atto647N. Scale bar, 2 µm. (b) Fraction of immobile trajectories of mGluR5WT (n = 8) and mGluR5ΔC (n= 11; p = 0.0004, two-sided unpaired- t-test). (c) Fraction of mobile trajectories with transient confinement zones for mGluR5WT (n = 8) and mGluR5ΔC (n = 11; p < 0.0001, two-sided unpaired t-test). (d) Relative frequency distributions of D_{effs} of individual mGluR5WT and mGluR5ΔC trajectories inside confinement zones. (e) Relative frequency plot of the average radius of confinement zones for mGluR5WT and mGluR5ΔC trajectories. Data are represented as means ± SEM. ***p < 0.001. Source data are provided as a Source Data file.

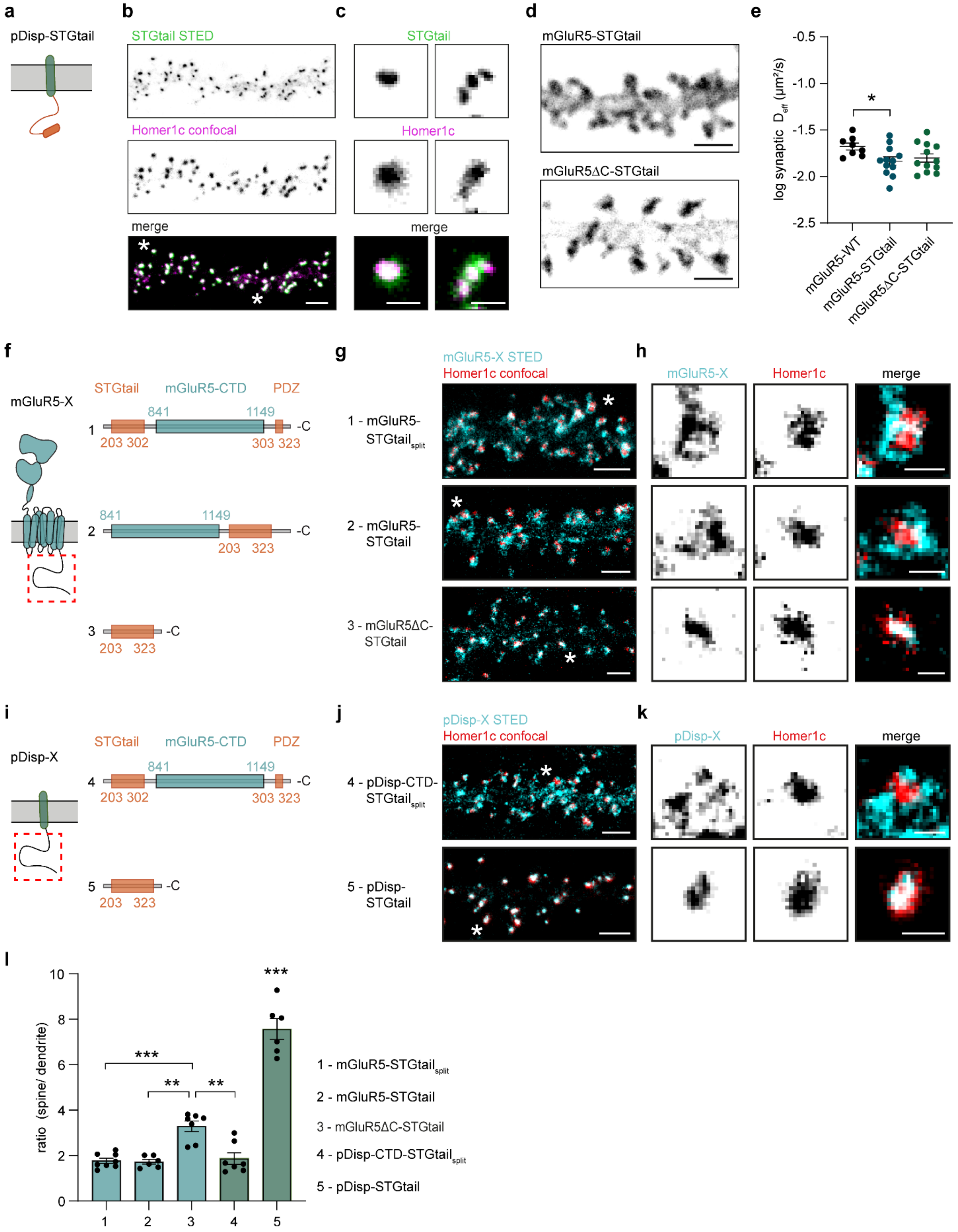
a
pDisp-STGtail
b
STGtail STED
Homer1c confocal
merge
c
STGtail
Homer1c
merge
d
mGluR5-STGtail
mGluR5ΔC-STGtail
e
log synaptic D_{eff} (µm²/s)
-0.5
-1.0
-1.5
-2.0
-2.5
*
mGluR5-WT
mGluR5-STGtail
mGluR5ΔC-STGtail
f
mGluR5-X
STGtail
mGluR5-CTD
PDZ
841
1149
203 302
303 323
203 323
-C
g
mGluR5-X STED
Homer1c confocal
1 - mGluR5-STGtail split
2 - mGluR5-STGtail
3 - mGluR5ΔC-STGtail
h
mGluR5-X
Homer1c
merge
i
pDisp-X
j
pDisp-X STED
4 - pDisp-CTD-STGtail split
5 - pDisp-STGtail
k
pDisp-X
l
ratio (spine/ dendrite)
***
**
1 - mGluR5-STGtail split
2 - mGluR5-STGtail
3 - mGluR5ΔC-STGtail
4 - pDisp-CTD-STGtail split
5 - pDisp-STGtail

Figure S6. The mGluR5-Stargazin chimera is efficiently targeted to the PSD

(a) Schematic of pDisp-TM-STGtail. (b) Representative gSTED image of dendrite expressing SEP-TM-STGtail, additionally labeled with an anti-GFP nanobody Atto647N (green), and Homer1c-mCherry (magenta; confocal). Scale bar, 2 µm. This experiment was replicated in cultures from more than 3 independent preparations of hippocampal neurons. (c) Zooms of spines indicated in *b* with asterisks. Scale bar, 500 nm. (d) Representative confocal images of dendrite with SEP-mGluR5-STGtail (top) and SEP-mGluR5ΔC-STGtail (bottom) expression, surface-labelled with an anti-GFP nanobody Atto647N. Scale bar, 2 µm. (e) Mean log D_{eff} per neuron of synaptic trajectories of mGluR5WT (n = 8), mGluR5-STGtail (n = 12) and mGluR5ΔC-STGtail (n = 12; p = 0.0705, one-way ANOVA with Dunnet's multiple comparisons test: compared to WT p = 0.0459 for mGluR5-STGtail and p = 0.1270 for mGluR5ΔC-STGtail). The mGluR5WT dataset shown *e* is also shown in *Figure 5g*, as these figures show different aspects of the same experiment. (f) Schematic of mGluR5 with the CTD domain structure of three variants: 1 – mGluR5-$STGtail_{split}$, 2 – mGluR5-STGtail and 3 – mGluR5ΔC-STGtail (g) Representative gSTED images of dendrite expressing mGluR5-X, additionally labeled with an anti-GFP nanobody Atto647N (cyan), and Homer1c-mCherry (red; confocal). Scale bar, 2 µm. (h) Zooms of spines indicated in *g* with asterisks. Scale bar, 500 nm. (i) Schematic of pDisp with the CTD domain structure of two variants: 4 – pDisp-CTD-$STGtail_{split}$ and 5 – pDisp-STGtail. (j) Representative gSTED images of dendrite expressing pDisp-X, additionally labeled with an anti-GFP nanobody Atto647N (cyan), and Homer1c-mCherry (red; confocal). Scale bar, 2 µm. (k) Zooms of spines indicated in J with asterisks. Scale bar, 500 nm. (l) Quantification of the ratio of spine over dendrite intensity of mGluR5-$STGtail_{split}$ (n = 8), mGluR5-STGtail (n = 6), mGluR5ΔC-STGtail (n = 7), pDisp-CTD-$STGtail_{split}$ (n = 7) and pDisp-STGtail (n = 6; p < 0.0001, one-way ANOVA with Tukey's multiple comparisons test: p > 0.9999 for 1 vs. 2, p = 0.0009 for 1 vs. 3, p = 0.0015 for 3 vs. 2, p = 0.9981 for 4 vs. 1, p = 0.0029 for 4 vs. 3 and p < 0.0001 for 5 vs. 1, 2, 3 and 4). Data are represented as means ± SEM. *p < 0.05, **p < 0.01 and ***p < 0.001. Source data are provided as a Source Data file.

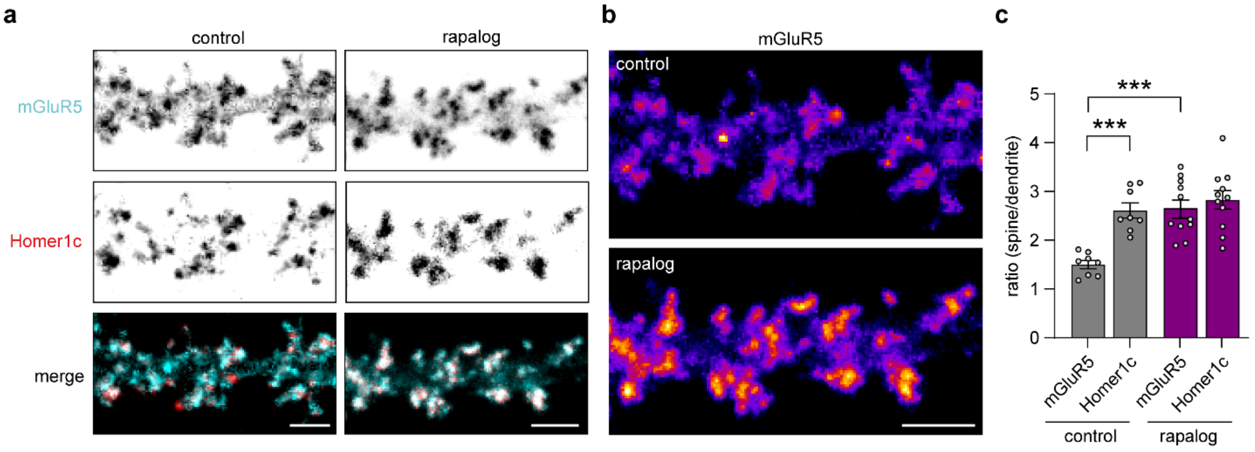

Figure S7. The inducible dimerization system acutely relocalized mGluR5 to the synapse

(a) Representative image of SEP-mGluR5-FRB and 2xFKBP-Homer1c-mcherry after vehicle (control) and rapalog application for 50 minutes. Scale bars, 2 µm. (b) SEP-mGluR5-FRB color-coded for fluorescence intensity in control and rapalog neurons. Scale bar, 2 µm. (c) Quantification of the ratio of spine over dendrite intensity of SEP-mGluR5-FRB and 2xFKBP-Homer1c-mCherry after vehicle (control; n = 8) and rapalog (n = 11) application for 50 minutes ($p < 0.0001$, one-way ANOVA with Tukey's multiple comparisons test: $p = 0.0002$ for mGluR5 control vs. mGluR5 rapalog, $p = 0.0007$ for mGluR5 control vs. Homer1c control, $p = 0.8598$ for mGluR5 rapalog vs. Homer1c rapalog and $p = 0.7935$ for Homer1c control vs. Homer1c rapalog). Data are represented as means ± SEM. ***$p < 0.001$. Source data are provided as a Source Data file.

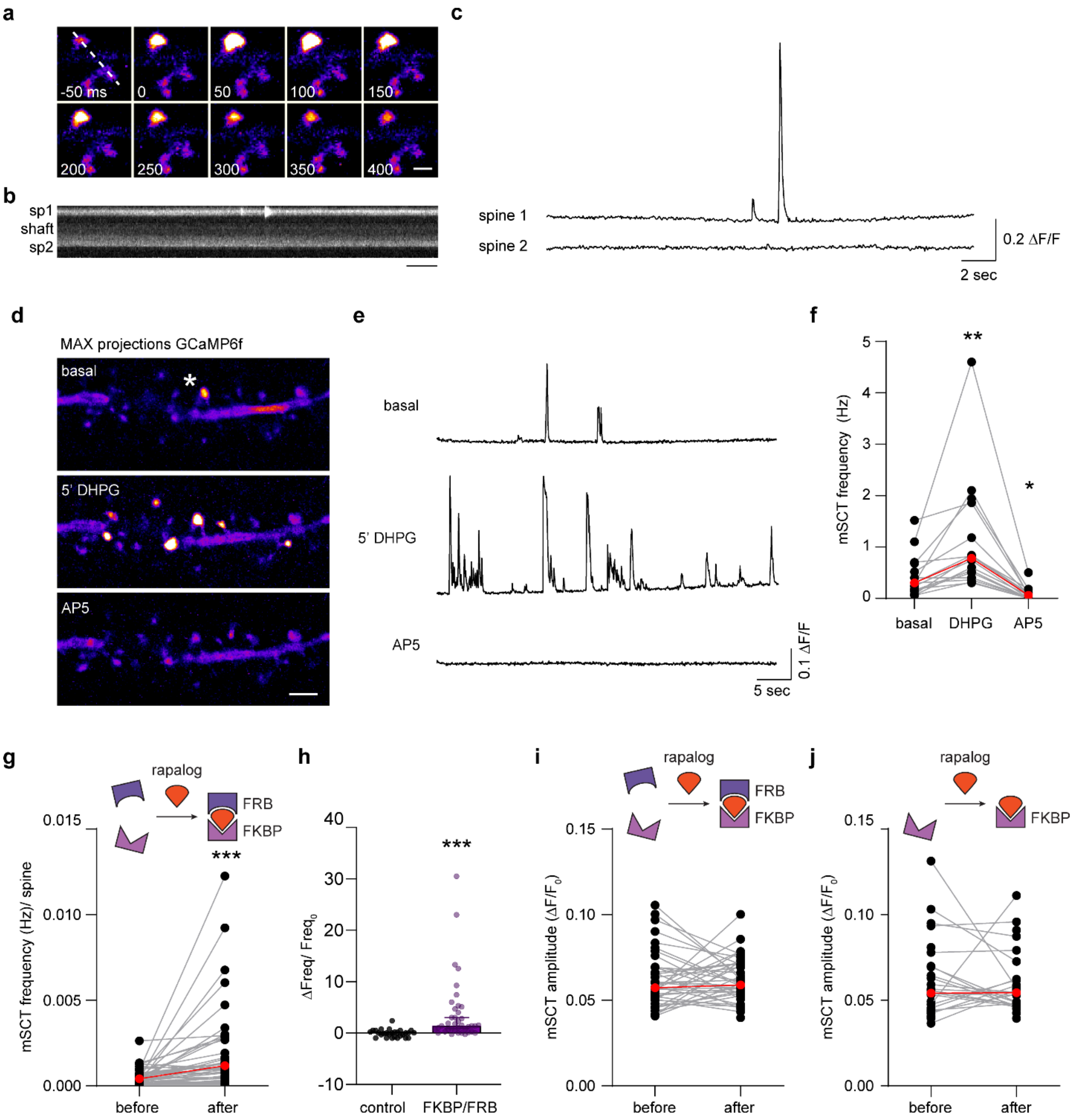

Figure S8. Synaptic recruitment of mGluR5 deregulated synaptic calcium signaling

(a) Representative time-lapse of a spine with GCaMP6f expression showing one mSCT (scale bar, 1 μm), and (b) the kymograph showing two consecutive events at the same spine (sp1), and no detected Ca^{2+} increases at the dendritic shaft and neighboring spine (sp2). Scale bar, 2 sec. This experiment was replicated in cultures from more than 3 independent preparations of hippocampal neurons. (c) The two successive mSCTs from spine 1 with different amplitudes and the absence of mSCTs in spine 2 (d) Maximum projections of the GCaMP6f stream (50 s) in a representative dendrite at baseline, upon 5 minute DHPG application and after the addition of AP5. Scale bar, 2 μm. (e) $\Delta F/F_0$ traces of GCaMP6f signal from

the spine indicated with an asterisk in *d* during baseline, upon DHPG and AP5 application. (f) Quantification of mSCT frequencies during baseline and after DHPG (5') and AP5 application in neurons without mGluR5 overexpression (n = 16 neurons, p < 0.0001, Friedman test with Dunn's multiple comparisons test: compared to basal p = 0.0094 for DHPG and p = 0.0207 for AP5). (g) Quantifcation of mSCT frequencies upon application of rapalog in neurons expressing SNAP-mGluR5-FRB and 2xFKBP-Homer1c-mCherry, corrected for spine number (n = 43 neurons, p < 0.0001, two-sided Wilcoxon matched-pairs signed rank test). (h) mSCT frequencies per neuron before and after application of rapalog for 30 minutes in control (FKBP only) and FKBP/FRB neurons. The scatter plot with bar (median with 95% CI) shows the change in mSCT frequency 30 minutes after rapalog application (ΔFreq), divided by the baseline value ($Freq_0$) (control: n = 28 and FKBP/FRB n = 39 neurons; p < 0.0001, two-sided Mann-Whitney test, only neurons included with minimum of one mSCT at baseline). (i) Quantification of mSCT amplitudes upon application of rapalog in neurons expressing SNAP-mGluR5-FRB and 2xFKBP-Homer1c-mCherry (n = 39 neurons, p = 0.9395, two-sided Wilcoxon matched-pairs signed rank test) and (j) in neurons expressing SNAP-mGluR5 and 2xFKBP-Homer1c-mCherry (control) (n = 22 neurons, p = 0.4628, two-sided Wilcoxon matched-pairs signed rank test). Medians are indicated by the red lines. . *p < 0.05, **p < 0.01 and ***p < 0.001. Source data are provided as a Source Data file.
