# Supplementary material for: mGluR5 is transiently confined in perisynaptic nanodomains to shape synaptic function
Source: Nat Commun. 2023 Jan 16;14:244. doi: 10.1038/s41467-022-35680-w (PMC9842668; doi:10.1038/s41467-022-35680-w)
Supplement: Supplementary file 3 — Reporting Summary [file 41467_2022_35680_MOESM3_ESM.pdf]

nature portfolio

Corresponding author(s): Harold D. MacGillavry

Last updated by author(s): 12-12-2022

Reporting Summary

Nature Portfolio wishes to improve the reproducibility of the work that we publish. This form provides structure for consistency and transparency in reporting. For further information on Nature Portfolio policies, see our Editorial Policies and the Editorial Policy Checklist.

Statistics

For all statistical analyses, confirm that the following items are present in the figure legend, table legend, main text, or Methods section.

n/a	Confirmed	
☐	☒	The exact sample size (*n*) for each experimental group/condition, given as a discrete number and unit of measurement
☐	☒	A statement on whether measurements were taken from distinct samples or whether the same sample was measured repeatedly
☐	☒	The statistical test(s) used AND whether they are one- or two-sided<br>*Only common tests should be described solely by name; describe more complex techniques in the Methods section.*
☒	☐	A description of all covariates tested
☐	☒	A description of any assumptions or corrections, such as tests of normality and adjustment for multiple comparisons
☐	☒	A full description of the statistical parameters including central tendency (e.g. means) or other basic estimates (e.g. regression coefficient) AND variation (e.g. standard deviation) or associated estimates of uncertainty (e.g. confidence intervals)
☐	☒	For null hypothesis testing, the test statistic (e.g. *F*, *t*, *r*) with confidence intervals, effect sizes, degrees of freedom and *P* value noted<br>*Give P values as exact values whenever suitable.*
☒	☐	For Bayesian analysis, information on the choice of priors and Markov chain Monte Carlo settings
☒	☐	For hierarchical and complex designs, identification of the appropriate level for tests and full reporting of outcomes
☒	☐	Estimates of effect sizes (e.g. Cohen's *d*, Pearson's *r*), indicating how they were calculated

Our web collection on statistics for biologists contains articles on many of the points above.

Software and code

Policy information about availability of computer code

Data collection	All data were collected using commerical software: NimOS version 1.6.1.9898 (ONI), MetaMorph version 7.10.2.240 (Molecular Devices), Micromanager (ImageJ) (Chazeau et al., 2016), Leica Application Suite X (LAS-X) software version 3.1.5.16308.
Data analysis	Data analysis was performed using ImageJ 1.52p (NIH; RRID: SCR_003070), Fiji (RRID: SCR_002285), GraphPad Prism 9 (RRID: SCR_002798), MATLAB R2019b (RRID: SCR_001622) and pCLAMP software with Clampfit 10.3 (Molecular Devices; RRID: SCR_011323). Figures were generated in Adobe Illustrator CC version 27.1.

For manuscripts utilizing custom algorithms or software that are central to the research but not yet described in published literature, software must be made available to editors and reviewers. We strongly encourage code deposition in a community repository (e.g. GitHub). See the Nature Portfolio guidelines for submitting code & software for further information.

Data

Policy information about availability of data

All manuscripts must include a data availability statement. This statement should provide the following information, where applicable:

- Accession codes, unique identifiers, or web links for publicly available datasets
- A description of any restrictions on data availability
- For clinical datasets or third party data, please ensure that the statement adheres to our policy

The data that support the findings of this study and Matlab code used for analysis are available from the authors upon request. Source data are provided with this paper.

Field-specific reporting

Please select the one below that is the best fit for your research. If you are not sure, read the appropriate sections before making your selection.

- [x] Life sciences
- [ ] Behavioural & social sciences
- [ ] Ecological, evolutionary & environmental sciences

For a reference copy of the document with all sections, see nature.com/documents/nr-reporting-summary-flat.pdf

Life sciences study design

All studies must disclose on these points even when the disclosure is negative.

Sample size	We did not perform formal power analysis to calculate sample sizes because the effect sizes were unknown prior to the study. Sample sizes were chosen based on our previous experience with similar experimental super-resolution microscopy datasets (see e.g. Bodzeta et al. MBoC 2022 and Catsburg et al., Elife 2022) and calcium measurements (e.g., Metzbower et al., eNeuro 2019). All the samples were drawn from sufficient independent experimental preparations (>3 independent cultures) and the sample sizes were sufficient judged from the reproducibility of the across independent experiments.
Data exclusions	For analysis of live cell data, neurons that died or if the focus was lost during image acquisition were excluded. SMLM and SMT data were excluded following predetermined criteria, such as localization precision and density, synaptic area and trajectory length, to ensure accurate determination of the dynamic subsynaptic organisation of the investigated proteins. Moreover, in some experiments we were specifically interested in a subset of localizations and trajectories of the investigated proteins which were selected following pre-established criteria based on overlap with a synaptic marker. For calcium imaging only spines clearly separated from the dendritc base and in focus were used for analysis. For the analysis of calcium transient amplitudes only spines with activity were included. For all experiments only neurons with similar expression levels of the investigated proteins were included in the study.
Replication	Each experiment was replicated in cultures from at least 3 independent preparations of hippocampal neurons. All replications were succesful.
Randomization	Randomisation is not relevant to this study as samples were not divided into experimental groups.
Blinding	For the analysis of gSTED localization data the experimenter was blinded to group allocations. For the majority of experiments, investigators were not blinded during data collection and analysis due to performing challenging and multi-step experimental procedures, which would make it technically complicated to do so as only one experimenter was sufficiently trained. However, data analysis in this study was performed automatically using software and required no user input, thus blinding was not necessary for this study.

Reporting for specific materials, systems and methods

We require information from authors about some types of materials, experimental systems and methods used in many studies. Here, indicate whether each material, system or method listed is relevant to your study. If you are not sure if a list item applies to your research, read the appropriate section before selecting a response.

Materials & experimental systems

n/a	Involved in the study	
☐	☒	Antibodies
☒	☐	Eukaryotic cell lines
☒	☐	Palaeontology and archaeology
☐	☒	Animals and other organisms
☒	☐	Human research participants
☒	☐	Clinical data
☒	☐	Dual use research of concern

Methods

n/a	Involved in the study	
☒	☐	ChIP-seq
☒	☐	Flow cytometry
☒	☐	MRI-based neuroimaging

Antibodies

Antibodies used	Rabbit anti-mGluR5 Polyclonal Antibody (Millipore; Cat# 06-451; RRID: AB_2313604), Rabbit anti-mGluR5 Polyclonal Antibody (Alomone Labs; Cat# AGC-007; RRID: AB_2039991), Mouse anti-c-Myc (9E10) Monoclonal Antibody (Santa Cruz Biotechnology; Cat# sc-40; RRID: AB_627268), Mouse anti-PSD-95 Monoclonal Antibody (Neuromab; Cat# 75-028; RRID: AB_2292909), Rabbit anti-GFP Polyclonal Antibody (MBL; Cat# 598; RRID: AB_591819), Phalloidin Alexa Fluor 594 (Thermo Fisher scientific; Cat# A12381; RRID: AB_2315633), Goat anti-Mouse IgG (H+L) Secondary Antibody, Alexa Fluor 488 (Thermo Fisher scientific; Cat# A11029; RRID: AB_138404), Goat anti-Rabbit IgG (H+L) Secondary Antibody, Alexa Fluor 488 (Thermo Fisher scientific; Cat# A11034; RRID: AB_2576217), Goat anti-Mouse IgG (H+L) Secondary Antibody, Alexa Fluor 594 (Thermo Fisher scientific; Cat# A11032; RRID: AB_2534091), Goat anti-Rabbit IgG (H+L) Secondary Antibody, Atto 647N (Sigma-Aldrich; Cat# 40839; RRID:AB_1137669), GFP-booster Atto647N (Chromotek; Cat#: gba647n; RRID: AB_2629215), Fluotag®-X4 anti-GFP Atto647N (Nanotag Biotechnologies; Cat# N0304-At647N-S; RRID: AB_2744632), Fluotag®-X4 anti-GFP Alexa Fluor 647 (Nanotag Biotechnologies; Cat# N0304-AF647-S; RRID: AB_2744632)

Validation

Rabbit anti-mGluR5 Polyclonal Antibody (Millipore; Cat# 06-451; RRID: AB_2313604): the manufacturer's website mentions that the antibody has been tested for Westerm Blot, Immunohistochemistry and has species reactivity for mouse and rat. References: PMID:17154259, PMID:20151362, PMID:21858817 and PMID:31597090.

Rabbit anti-mGluR5 Polyclonal Antibody (Alomone Labs; Cat# AGC-007; RRID: AB_2039991): the manufacturer's website mentions that the antibody is suitable for Western Blot, Immunohistochemistry, Immunoprecipitation, Live cell imaging, Immunocytochemistry, Indirect flow cytometry and has species reactivity for human, mouse and rat. Some of the relevant references are PMID: 35746896, PMID: 31473770, PMID: 30926797 and PMID: 30926797.

Mouse anti-c-Myc (9E10) Monoclonal Antibody (Santa Cruz Biotechnology; Cat# sc-40; RRID: AB_627268): this antibody is suitable for WB, IP, IF, IHC(P), FCM, ELISA and has species reactivity for mouse, rat, human, monkey, feline and canine. Some of the relevant references are PMID:16917820, PMID:20394056, PMID:23224860, PMID:24899714.

Mouse anti-PSD-95 Monoclonal Antibody (Neuromab; Cat# 75-028; RRID: AB_2292909): this antibody is suitable for IWB, ICC, IB, IHC, KO, IP, IGEM and has species reactivity for human, mouse and rat. Some of the relevant references are PMID:17185748, PMID:17651419, PMID:17670980, PMID:18072193.

Rabbit anti-GFP Polyclonal Antibody (MBL; Cat# 598; RRID: AB_591819): this antibody is suitable for ChIP, EM, ICC, IHC, IP and WB. This antibody reacts with GFP on Western blotting, Immunoprecipitation, and Immunocytochemistry. This antibody also detects GFP-tagged proteins expressed in mammalian cell on Western blotting, Immunoprecipitation, Immunocytochemistry and Immunohistochemistry. Some of the relevant references are PMID:21800305, PMID:23124836, PMID:23640820, PMID:25308109.

Phalloidin Alexa Fluor 594 (Thermo Fisher scientific; Cat# A12381; RRID: AB_2315633): phalloidin selectively stains F-actin and can be used in tissue sections, cell cultures or cell-free preparations according to the manufacturer's website. Some of the relevant references are PMID:27966429, PMID:28193690, PMID:28244368, PMID:30021164.

GFP-booster Atto647N (Chromotek; Cat#: gba647n; RRID: AB_2629215): target antigens are eCFP, CFP, mCerulean,eGFP, wtGFP, GFP S65T, AcGFP, TagGFP, tagGFP2, sfGFP, pHluorineYFP, YFP, Venus, Citrine and the GFP-booster has been tested by the manufacturer on immunofluorescence. Relevant references are PMID:27791980, PMID:27935478, PMID:30782781, PMID:31513013.

Fluotag®-X4 anti-GFP Atto647N (Nanotag Biotechnologies; Cat# N0304-At647N-S; RRID: AB_2744632) and Fluotag®-X4 anti-GFP Alexa Fluor 647 (Nanotag Biotechnologies; Cat# N0304-AF647-S; RRID: AB_2744632): Recognizes GFP (green fluorescent protein) and common GFP derivatives like EGFP, mEGFP, Sirius, tSapphire, Cerulean, eCFP, mTurquoise, acGFP, Emerald, superecliptic pHluorin, paGFP, superfolder GFP, eYFP, mVenus and Citrine. The manufacturer mentions that the Fluotag ®-X4 anti-GFP nanobodies are suitable for immunofluorescence applications.

Animals and other organisms

Policy information about studies involving animals; ARRIVE guidelines recommended for reporting animal research

Laboratory animals

2.5 months old female pregnant Wistar rats were obtained from Janiver. Hippocampal cultures were prepared from embryonic day 18 (E18) rat brains of both genders.

Wild animals

This study did not involve wild animals.

Field-collected samples

This study did not involve samples collected from the field.

Ethics oversight

All animal experiments were performed in compliance with the guidelines for the welfare of experimental animals issued by the Government of the Netherlands (Wet op de Dierproeven, 1996) and European regulations (Guideline 86/609/EEC). All animal experiments were approved by the Dutch Animal Experiments Review Committee (Dier Experimenten Commissie; DEC), performed in line with the institutional guidelines of Utrecht University.

Note that full information on the approval of the study protocol must also be provided in the manuscript.
